# Diagnosis and Management of Spontaneous Intracranial Hypotension Due to a CSF Leak: A Case of Spontaneous Recovery

**DOI:** 10.7759/cureus.77960

**Published:** 2025-01-25

**Authors:** Hana Elamin, M. Nabeel Pervez, Fraser Gordon

**Affiliations:** 1 General Medicine, University Hospitals of Morecambe Bay, Lancaster, GBR; 2 General Medicine, Royal Lancaster Infirmary, Lancaster, GBR; 3 Geriatrics, University Hospitals of Morecambe Bay, Lancaster, GBR; 4 Stroke, Royal Lancaster Infirmary, Lancaster, GBR

**Keywords:** bony spur, headache, spontaneous csf leak, spontaneous intracranial hypotension (sih), stroke

## Abstract

Spontaneous intracranial hypotension (SIH) is a rare condition typically caused by a CSF leak at the level of the spine, leading to a reduction in intracranial pressure (ICP). This case describes a 55-year-old man who presented with visual disturbance, intermittent occipital headaches, nausea, altered hearing, and unsteady gait. The initial MRI of the head showed shallow bilateral subdural hematomas, which were believed to be secondary to the stretching of subdural veins. A diagnosis of probable intracranial hypotension was made and further radiological imaging of the spine was carried out to look for the presence of a CSF leak. A CT myelogram identified a CSF leak in the anterior part of the dura at the T1/T2 level, which was believed to be secondary to a bony spur. There are no well-defined statistics on the incidence of the causes of SIH. After a multidisciplinary discussion, a targeted CT-guided epidural blood patch was planned; however, the patient reported improvement in symptoms, so the procedure was abandoned, with serial follow-ups advised.

## Introduction

The intracranial pressure (ICP) is also known as the CSF pressure in a prone position, given the equilibrium between the CSF secretion, absorption, and resistance to flow in this position [1]. The normal ICP ranges from 10 to 15 mmHg in adults; this can be measured invasively by using a pressure transducer connected to the CSF spaces via an external lumbar drain or ventricular drain [1]. It can also be estimated non-invasively using MRI scans. While our bodies are excellent when it comes to maintaining a normal ICP, there are a lot of conditions that disrupt this normal physiology.

A low ICP, also known as intracranial hypotension, is a clinical syndrome resulting from low CSF volume [2]. This condition can be caused by a congenital defect in the dura, allowing CSF to escape, trauma to the dura (which can occur after spinal surgery or a lumbar puncture), or implanted shunts placed to treat CSF accumulation [3].

This condition affects both genders and various age groups but is diagnosed more frequently in females. Approximately four in 100,000 individuals are diagnosed with spontaneous intracranial hypotension (SIH), if not more [4].

Patients with intracranial hypotension usually present with orthostatic headache. In severe cases, nausea, vomiting, and photophobia can be seen, and rarely, decreased level of consciousness [2].

## Case presentation

A healthy 55-year-old gentleman with no medical history presented to the hospital with visual disturbances in both eyes and balance problems over the preceding 24 hours. At the time of presentation, he reported developing an intermittent headache two weeks prior, located in the occipital area and moderate in severity, but not associated with any neurological deficits. Physical examination revealed equal and reactive pupils, no nystagmus, but double vision in both eyes. Additionally, the patient was unsteady during the tandem gait test.

**Figure 1:**
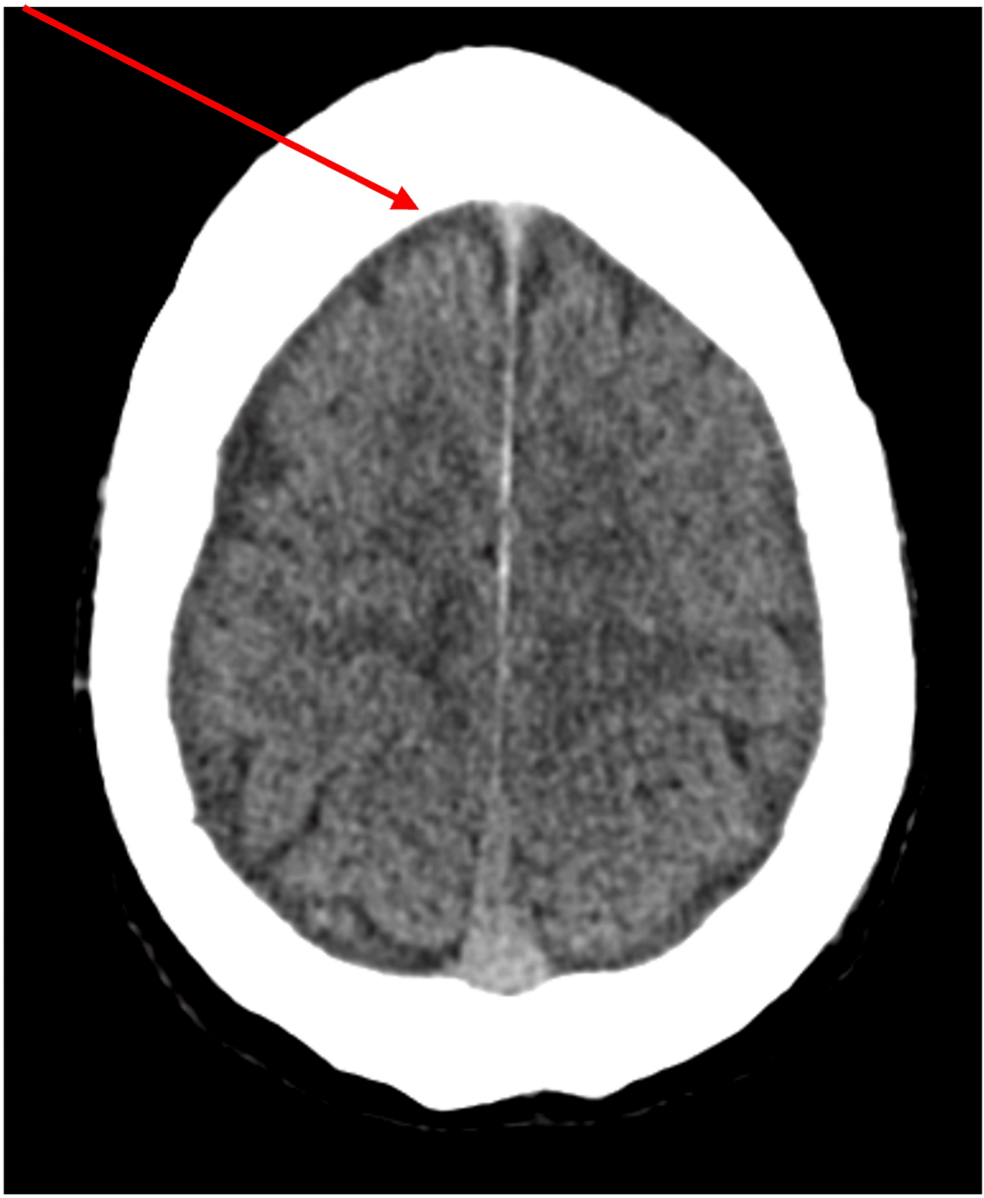
CT head showing the prominence of the subdural spaces bilaterally within the vertex overlying the frontal lobes

An initial CT head showed mild bilateral subdural hygromas (Figure 1). This was reviewed and an MRI of the brain was undertaken for further evaluation. 

**Figure 2:**
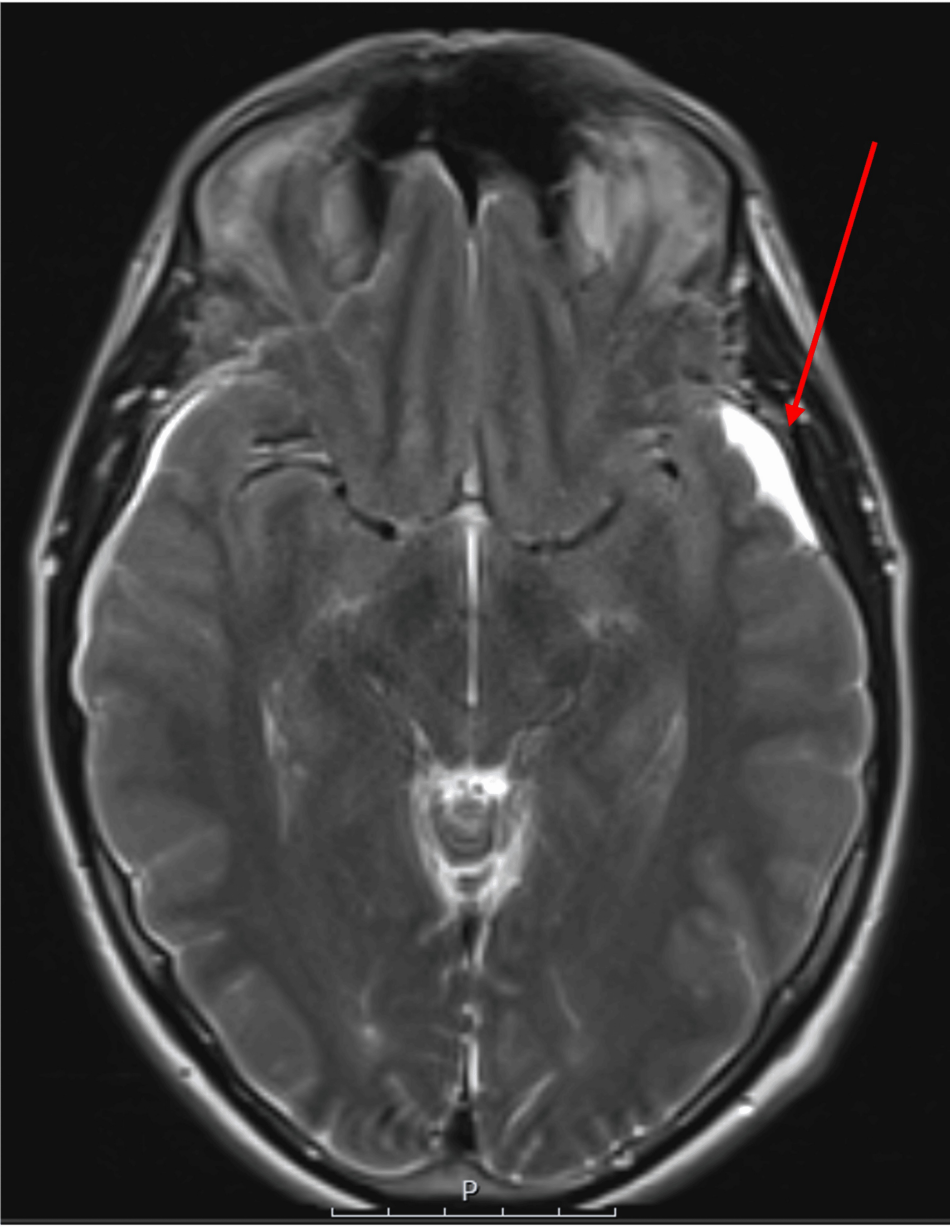
MRI of the head showing bilateral shallow subdural hematomas

**Figure 3:**
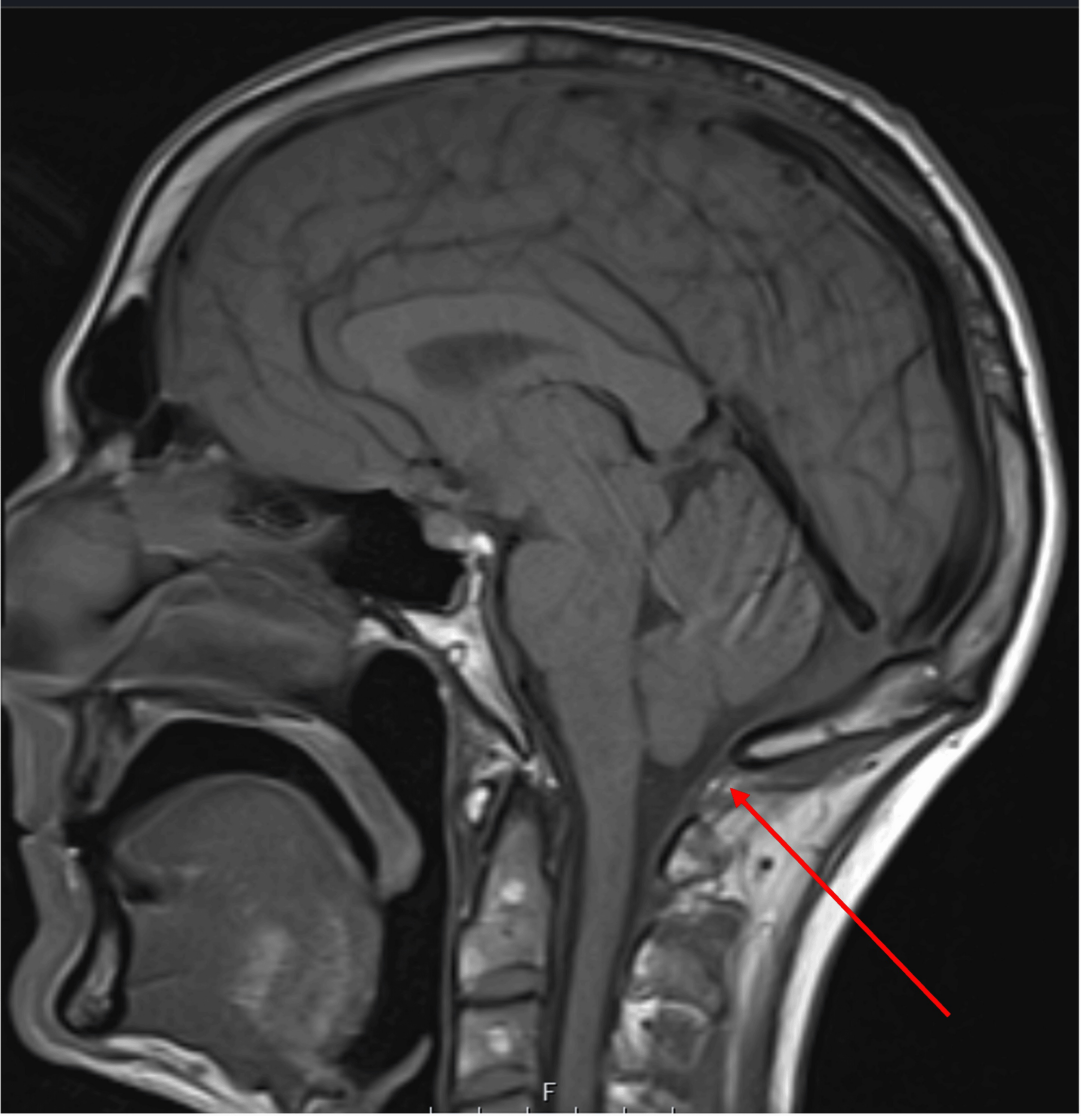
MRI of the head showing slight herniation of the cerebellar tonsils

The MRI of the brain showed shallow bilateral subdural hematomas (Figure 2) secondary to apparent stretching of subdural veins, despite the patient denying any history of head injury or trauma. This, along with a slit-like ventricular system, slight herniation of the cerebellar tonsils (Figure 3), the rounded appearance of the adenohypophysis, and relative effacement of the prepontine cistern, is consistent with a presentation of intracranial hypotension.

**Figure 4:**
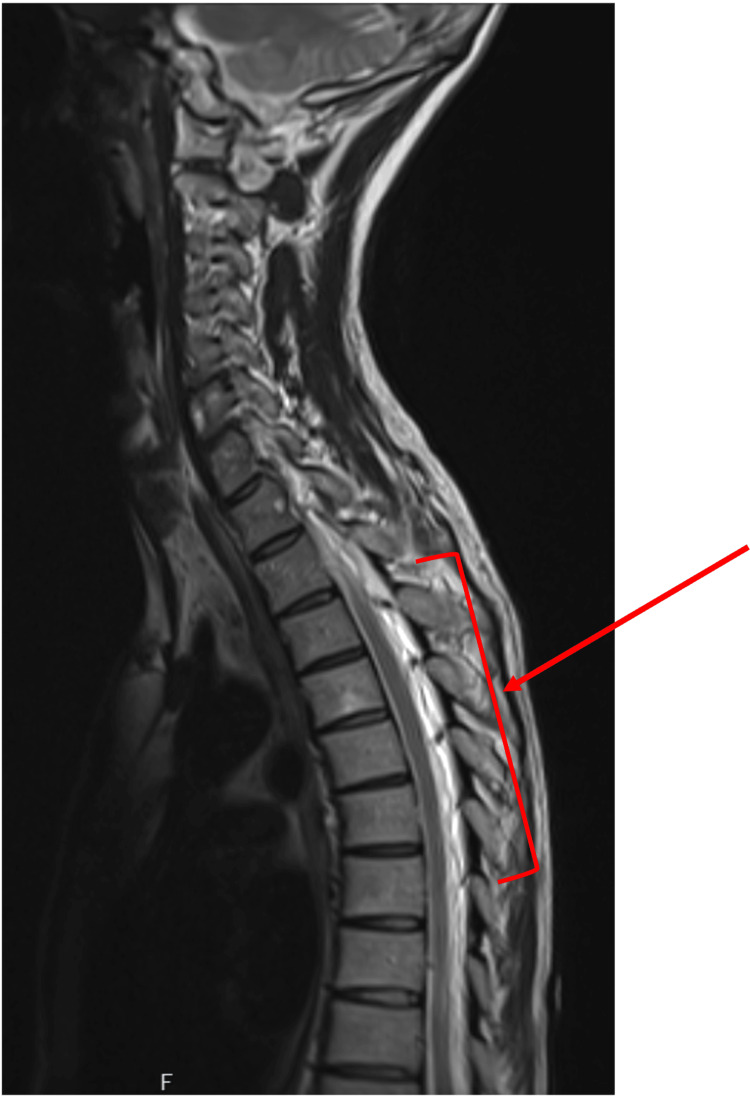
MRI of the whole spine showing congestion and tortuosity of the posterior epidural veins extending from the T2/T3 level down to the T8/T9 level

The scan results were discussed with the regional neurosurgical center, and an MRI of the spine was performed to identify the focus of any CSF leak. The spinal MRI showed distension and tortuosity of the posterior epidural veins, suggesting congestion extending from T2/T3 to T8/T10 (Figure 4). The patient was transferred to the regional neurosurgical center for further evaluation.

**Figure 5:**
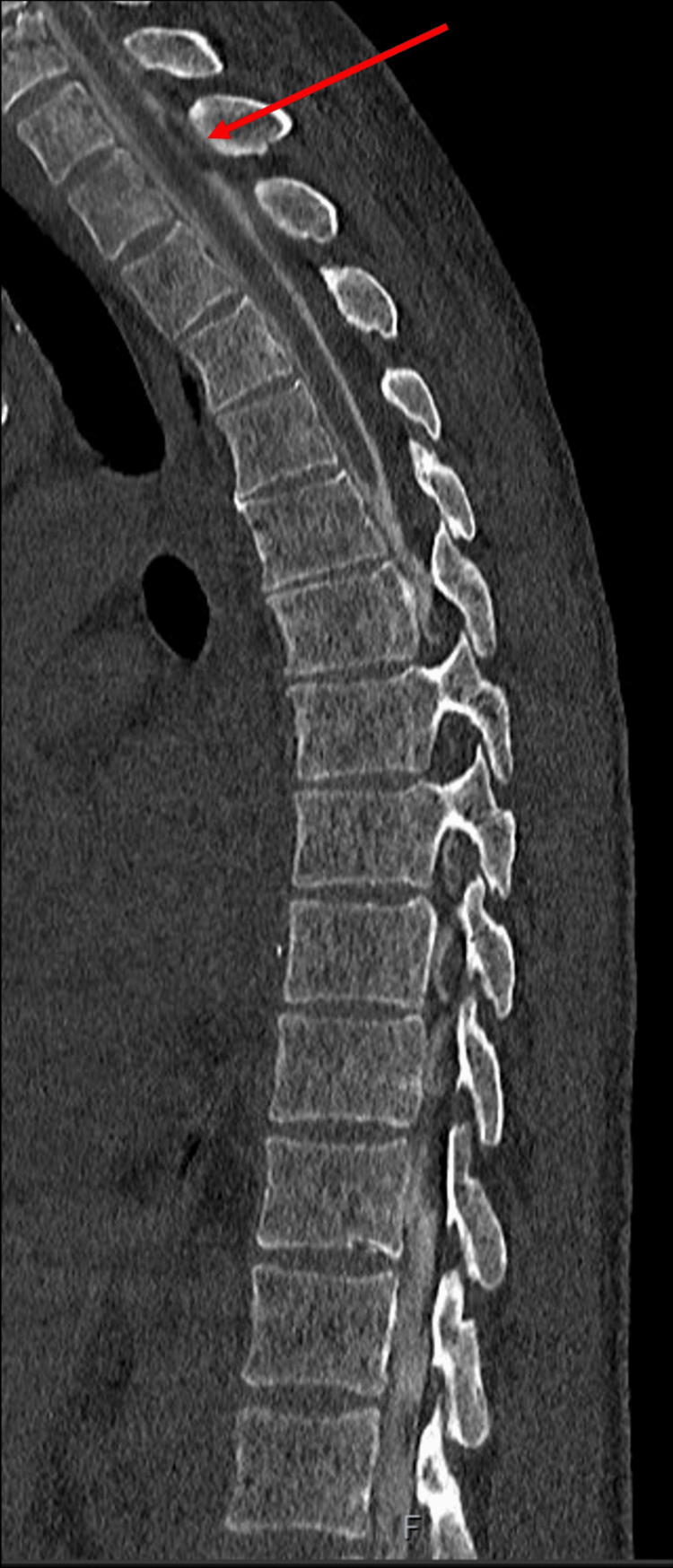
Dynamic CT myelogram showing a thin longitudinal extradural fluid collection along the dorsal aspect of the thecal sac in the cervical and upper thoracic regions

**Figure 6:**
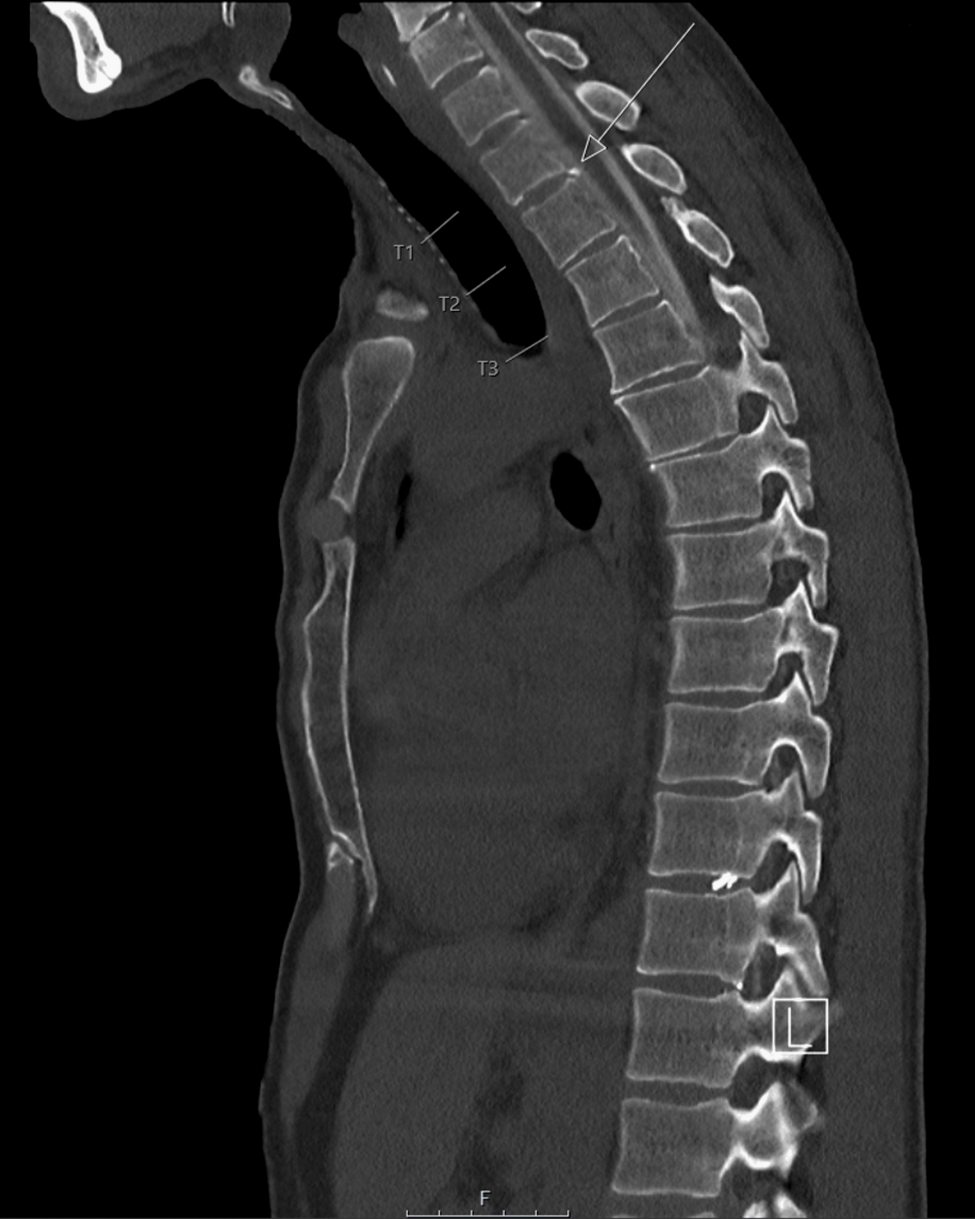
CT spine myelogram showing an arrow pointing to a small calcified, pointed osteophyte at the T1-T2 level

In the following week, the patient underwent dynamic CT myelograms in the Trendelenburg position, which did not identify any CSF leak. Another dynamic CT myelogram was performed in the right lateral decubitus position, which identified a thin longitudinal extradural fluid collection along the dorsal aspect of the thecal sac in the cervical and upper thoracic region (Figure 5), which was thought to be secondary to a bone spur causing the dural tear (Figure 6). A CT-guided epidural blood patch was initially planned following a discussion with the neuro-interventional radiologists; however, the patient reported that his symptoms were improving and had almost fully resolved. Given the full resolution of symptoms, there was no indication of a blood patch, and the patient was discharged.

## Discussion

SIH is a lesser-known disorder evidenced by its frequency of presentation. A four-year observational study between 2002 and 2006 puts the incidence rate at five per 100,000 [5]. A further population-based incidence study over a period of 14 years (2006 to 2020) found the average annual incidence of SIH for all age groups at 3.7 per 100,000 population [6]. These studies show that while not rare, it is nonetheless an uncommon disorder [6]. A German research article has reported an increasing number of referrals and subsequent treatment at their neurosurgery center for SIH [4]. However, while this incidence is comparable to other uncommon diseases such as idiopathic intracranial hypertension, trigeminal neuralgia, and cluster headaches [6], it is still lesser known among healthcare professionals [7], and epidemiologic data are scarce, with the overall prevalence remaining unknown [8]. Our case adds to the available literature.

In terms of what causes SIH, the factors or predisposing conditions listed in the available literature include disc degenerative disease and connective tissue disorders, which make the meningeal layers prone to tearing or diverticula [9]. Trauma, even minor like sneezing, has also been implicated [10]. According to a classification system, CSF leaks are mainly three types: Type 1 is due to dural tears, type 2 is due to meningeal diverticula, and type 3 is due to a CSF venous fistula. This index study included 588 patients and the incidence of each kind of leak has been put at 26.6%, 42.3%, and 28.7% for types 1, 2, and 3, respectively [11]. As is evident from this comprehensive study, dural tears as a cause of SIH is the least common and our case report has added to the available literature for cause of SIH. 

Although the incidence of dural tears has been documented in the case study mentioned, the specific incidence of the exact underlying causes (among which is a bony spur) has not, to the best of our knowledge, been reported.

In our case, the patient underwent dynamic CT myelograms to identify the cause of the leak, and after a multidisciplinary team discussion, a targeted epidural patch was recommended (which the patient ultimately did not undergo). This approach differs from the consensus guidelines [12,13] but tallies with the approach suggested by Farnsworth et al. [14]. The guidelines suggest starting with conservative treatment, followed by a non-targeted epidural blood patch, then multidisciplinary team (MDT) discussions and invasive investigations, such as dynamic CT myelogram, to isolate the site of the leak before offering a targeted epidural blood patch. As mentioned, guidelines for the management of SIH were only recently produced, with the studies cited from 2023 and 2024. Prior to that, there were no dedicated guidelines. We have also made a reference to another study where a different approach was taken, which belongs to 2023 as well. We are still in the nascent stages with regard to guidelines for SIH. Dedicated guidelines for SIH will increase awareness of its management as well. The different approach in our case highlights the importance of case-by-case discussion in a multidisciplinary setting and individualized care. 

In terms of treatment, a conservative approach is an available option. Based on symptoms, patients may be offered conservative treatment only initially. The success of conservative treatment alone has been put at 28% as opposed to 64% for a single epidural blood patch by one meta-analysis; this meta-analysis included 144 articles with a mean of 53 patients with SIH in each [15]. Conservative management is not the most practical [14] as it entails complete bed rest. Our patient was quite symptomatic and as a result, went on to have a dynamic myelogram with a plan for a targeted epidural blood patch and further suggestions for surgery as well.

However, over a period of three months (the patient was admitted at the start of February and was scheduled to undergo a targeted epidural patch in April after MDT discussions and dynamic myelograms), his symptoms resolved spontaneously. It was then decided that the patient would be followed up with MRI imaging in three months. This highlights how clinical management can change based on symptoms; however, follow-up remains integral.

## Conclusions

This case highlights the diagnostic challenges of SIH, often delayed due to its variable presentation and limited awareness, as well as the uncommon etiology underlying this condition. Additionally, it demonstrates that, in rare instances, SIH symptoms can resolve spontaneously without the need for active intervention.

To the best of our knowledge and based on our literature search, the exact number of CSF leaks caused by bone spurs is not well documented, likely because it is a less common cause compared to other factors such as trauma, surgery, or spontaneous leaks. This case report aims to raise awareness among healthcare professionals of this presentation, so it can be considered in their differential diagnoses. Additionally, it demonstrates that a spinal bone spur can cause a dural tear and lead to SIH, which, as discussed above, is an uncommon cause. The importance of multidisciplinary care is also highlighted, with stroke, neurosurgery, and interventional neuroradiology all being involved.
